# Lipidomic profiling identifies key pathways and a 5-lipid panel with high diagnostic efficacy for ischemic stroke

**DOI:** 10.1038/s41598-026-42918-w

**Published:** 2026-03-05

**Authors:** Junhua Lu, Yuan Liu, Zhaoran Guan, Yue Wu, Ying Zhao, Ying Lin, Liqiu Ma, Ping Xue, Hongjun Guan

**Affiliations:** 1 1Department of Nursing, Mudanjiang Medical University, Mudanjiang, 157011 Heilongjiang P.R. China; 2 2Department of Public Health, Mudanjiang Medical University, Mudanjiang, 157011 Heilongjiang P.R. China; 3 College of Stomatology, Central South University, Changsha, 410000 Hunan P.R. China; 4 4Hongqi Hospital Affiliated to Mudanjiang Medical University, Mudanjiang, 157011 Heilongjiang P.R. China

**Keywords:** Ischemic stroke, Biomarkers, LC-MS, Lipid metabolism, Metabolic Pathways, Biochemistry, Biomarkers, Computational biology and bioinformatics, Diseases, Neurology, Neuroscience

## Abstract

Ischemic stroke (IS) accounts for over 80% of all stroke cases, presenting as a prevalent, debilitating cerebrovascular disorder with limited therapeutic options. The urgent need for early diagnostic biomarkers and insights into pathogenesis has highlighted dysregulated lipid metabolism as a key contributor, while metabolomics advances enable novel biomarker exploration. This study integrated bioinformatics and a case-control design to investigate IS-related lipid metabolism pathways and blood lipid biomarkers. Gene Expression Omnibus (GEO) gene expression datasets were analyzed via Gene Set Enrichment Analysis (GSEA) to identify lipid pathways, and case-control analyses employed Chi-square/Z tests for conventional blood lipids, Liquid Chromatography-Mass Spectrometry (LC-MS) for plasma small-molecule lipids, and orthogonal partial least squares discriminant analysis, t-tests, and Receiver Operating Characteristic (ROC) curves for validation. Results revealed five significantly downregulated lipid pathways (α-linolenic acid, linolenic acid, ether lipid, glycerophospholipid, and sphingolipid metabolism). IS patients exhibited dyslipidemia (elevated TC/TG/LDL-C, reduced HDL-C). Additionally, 15 differentially expressed lipid molecules were identified in a validation cohort after excluding the influence of comorbidities. Among these, five representative lipids (e.g., PE(P-18:1/22:4)) demonstrated potential diagnostic performance, with an area under the receiver operating characteristic curve (AUC) of 0.917, sensitivity of 60.0%, and specificity of 96.7%, indicating their potential utility as biomarkers for the early detection of IS.

Stroke is the second leading cause of death worldwide, following ischemic heart disease, and the third leading cause of disability, following neonatal disorders and ischemic heart disease^1^. It is characterized by high incidence, mortality, disability rate, and recurrence risk, often resulting in rapid neurological deterioration with severe consequences. Stroke primarily consists of hemorrhagic stroke (HS) and IS, with IS representing more than 80% of all cases and exhibiting a significantly higher prevalence than HS^2^. The occurrence of IS is influenced by multiple factors, including hypertension, hyperlipidemia, diabetes, smoking, and obesity^3^. Currently, recombinant tissue plasminogen activator (rt-PA) is the only approved pharmacological treatment for acute IS, primarily used for intravenous thrombolysis. However, its clinical utility is constrained by a narrow therapeutic time window and the risk of inducing symptomatic intracranial hemorrhage^4^. At present, the diagnosis of IS mainly relies on typical clinical symptoms combined with imaging examinations^5^. However, approximately 50% of IS patients lack specific imaging findings in the early stages^6^. Furthermore, the molecular mechanisms underlying IS are not fully understood, and no validated blood-based biomarkers are currently available for routine clinical use. Therefore, identifying novel biomarkers could enhance our understanding of IS etiology and pathophysiology and facilitate earlier diagnosis and timely intervention.

Lipids and their metabolites play a crucial role in maintaining the normal structure and function of the central nervous system^7^. During the pathological process of IS, maintaining normal brain function and metabolic homeostasis is particularly critical. Various lipid components, including simple lipids, phospholipids, glycolipids, and fatty acids, are not only essential nutrients for brain function but also important components of cell membrane structure^8^. Following cerebral ischemia, the reduction in cerebral blood flow disrupts systemic lipid homeostasis^9^. Extensive research has established a strong association between abnormal lipid metabolism and the onset and progression of IS, with dyslipidemia recognized as a major modifiable risk factor for stroke^10^. Research indicates that elevated levels of LDL-C increase the risk of IS^11^, while elevated levels of HDL-C may help reduce the risk of IS, especially in transient ischemic attack (TIA)^12^.

Given the high phenotypic diversity and genetic correlation among different lipid types^13^, the specific association between lipids and IS and its mechanism of action remain unclear. Therefore, clarifying the relationship between different lipid molecules and the risk of IS has significant public health and clinical significance. Metabolomics, as an important branch of systems biology, mainly studies the dynamic changes of metabolites during metabolic processes to reveal the metabolic characteristics of life activities. The detection results of metabolites can more accurately and intuitively reflect the pathological and physiological state of the body. Currently, blood and urine are the main sample sources for metabolomics research, and proteins, sugars and other impurities are usually removed to improve the accuracy of detection.

Metabolic disorders are regarded as one of the significant factors in the occurrence and development of IS. With the advancement of novel analytical techniques such as metabolomics and lipidomics, new research directions have been provided for identifying key lipid metabolic biomarkers with potential diagnostic and prognostic value.

In this study, we collected lipid profile indicators of IS patients and the control group, and analyzed the influence of traditional lipid parameters on IS. Meanwhile, we used LC-MS technology to detect lipid metabolites in serum samples of IS patients and healthy controls, and screened out metabolites with significant differences, with the aim of providing potential biomarkers for the early diagnosis of IS.

## Patients and methods

### Database collection and bioinformatics analysis

Peripheral blood gene expression profile data from patients with IS and healthy controls were retrieved from the GEO database (https://www.ncbi.nlm.nih.gov/geo/). After rigorous screening, relevant datasets that met the research criteria were downloaded.

The multiple datasets included in this study were merged using R software. The batch effect was corrected utilizing the “SVA R Package”^14^ to eliminate technical bias. Differential expression analysis was performed using the “Limma R Package”^15^, resulting in the identification of differentially expressed genes (DEGs). The screening criteria were established as *P* < 0.05 and |log fold change (FC)| > 1^16^ (A |log₂FC| > 1 can ensure that the magnitude of gene expression changes has biological significance and effectively filter out false positive results caused by minor expression fluctuations.).

Functional enrichment analysis for Gene Ontology (GO) and Kyoto Encyclopedia of Genes and Genomes (KEGG) pathways was conducted using the “clusterProfiler R Package” within R software^17^. Additionally, KEGG gene sets associated with lipid metabolism were obtained through the MSigDB database (http://www.gsea-msigdb.org/gsea/msigdb), followed by an evaluation of these gene sets enrichment degree among DEGs in IS patients via GSEA^18^, thereby identifying key regulatory genes.

### Study participants and ethics approval

This study employed a two-stage lipidomics research design comprising a discovery phase in a small cohort followed by validation in a larger cohort. All participants were recruited from Hongqi Hospital of Mudanjiang Medical University between October 2021 and October 2023. The study was approved by the Medical Ethics Review Committee of Mudanjiang Medical University (Approval No. 2021-s19) and conducted in accordance with the principles of the Declaration of Helsinki, as well as relevant national and international ethical guidelines and regulatory requirements. Written informed consent was obtained from all participants or their legal representatives prior to enrollment.

The study design included a discovery cohort and an internal validation cohort, with the former constituting a randomly selected subset of the latter. This approach facilitated an unbiased identification of differentially expressed lipids in a homogeneous population during the discovery phase, followed by robust validation in a larger sample, thereby minimizing the risk of overfitting while ensuring a balance between exploratory depth and statistical rigor.

### Internal validation cohort

(1) A total of 251 patients with IS, newly diagnosed in the Department of Neurology at Hongqi Hospital, Mudanjiang Medical University, between October 2021 and October 2023, were enrolled as the case group. The inclusion criteria were as follows: ①Patients with ischemic stroke whose time from onset to admission was no more than 48 h. The selected cases must meet the clinical symptoms and signs criteria for ischemic stroke established by the World Health Organization (WHO)^19^. ②The cases should be diagnosed as ischemic stroke through CT or MRI scans^20^. ③Age ≥ 18 years. ④Participants must have the willingness to participate in this study and be capable of signing the informed consent form. ⑤Patients with neurological abnormalities caused by trauma, metabolic disorders, toxin exposure, and tumors, as well as those with cerebral hemorrhage, subarachnoid hemorrhage, and transient ischemic attack, were excluded. ⑥Patients with a history of taking lipid-lowering drugs or other drugs that affect lipid levels within the past 3 months were excluded.

(2) A total of 251 healthy individuals who underwent routine physical examinations at the same hospital during the study period were enrolled as the control group. The inclusion criteria were as follows: ①No history of any cardiovascular or cerebrovascular diseases, including ischemic stroke, hemorrhagic stroke, subarachnoid hemorrhage, transient ischemic attack, coronary heart disease, myocardial infarction, atrial fibrillation, peripheral vascular disease, etc. ②No family history of any cardiovascular or cerebrovascular diseases. ③No history of any tumors. ④No history of any kidney diseases, liver diseases, or autoimmune diseases. ⑤Matched with the case group in terms of gender, age, and ethnicity. ⑥Excluded individuals with a history of taking lipid-lowering drugs or other drugs that affect lipid levels within the past 3 months.

### Discovery cohort

In the internal validation cohort, 15 newly diagnosed IS patients and 15 age- and gender-matched healthy controls were selected via simple random sampling to constitute the discovery cohort, excluding individuals with a history of medication use, unhealthy dietary habits, or other potential confounding factors that could affect lipid metabolism testing.

**Fig. 1:**
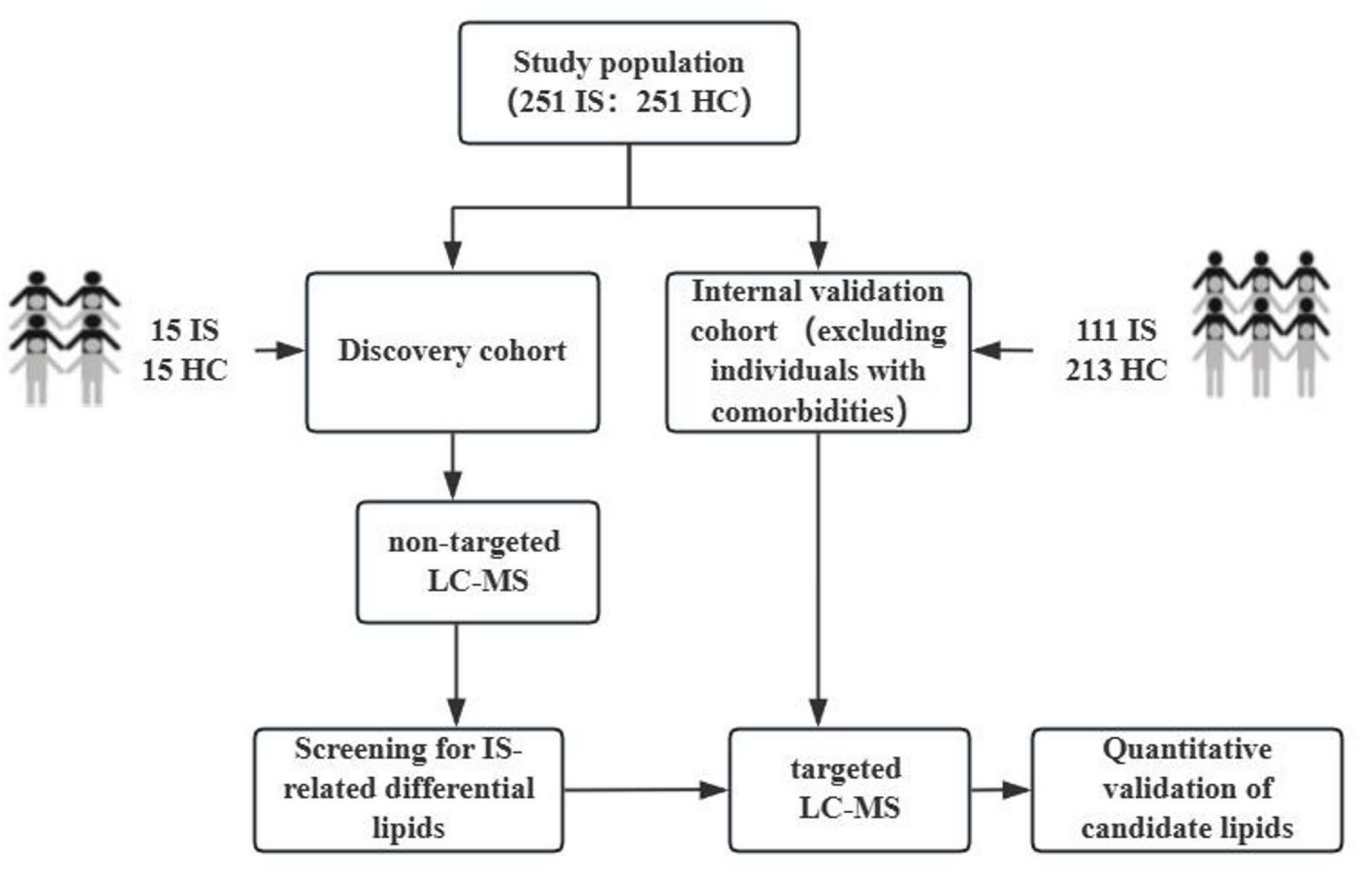
Research design.

The discovery cohort employed non-targeted liquid chromatography-mass spectrometry (LC-MS) to identify differentially expressed lipids associated with IS onset. Subsequently, the internal validation cohort utilized targeted LC-MS for precise quantification of selected candidate lipids, thereby ensuring the reliability of the findings and their potential for clinical translation. The research design is shown in Fig. 1.

### Sample collection

For all selected acute ischemic stroke patients and healthy controls, a unified record form was used to collect relevant information, including general data (gender, age), lipid parameters (TC, TG, HDL-C, LDL-C), and medication history (whether lipid-lowering drugs or other drugs were taken in the past 3 months).

All subjects in the experimental group fasted for 8–12 h and had 4 ml of fasting venous whole blood collected the next morning after admission and placed in 5 ml EDTA vacuum anticoagulant tubes. The control group fasted for 8–12 h and had 4 ml of fasting venous whole blood collected in the morning of the physical examination and placed in 5 ml EDTA vacuum anticoagulant tubes. Plasma and blood cells were separated and stored at -80 °C in ultra-low temperature freezers.

### Plasma sample pretreatment

Plasma samples were retrieved from the − 80℃ freezer and gradually thawed at 4℃. Subsequently, 180 µL of the sample was combined with 720 µL of a 1:1 acetonitrile/methanol (ACN/MeOH) solution. After being placed on ice for 2 min, the mixture was centrifuged at 4℃ and 20,000 rpm for 10 min. The supernatant was collected, and this process was repeated twice more. The supernatants from all three rounds were pooled together and evaporated under nitrogen gas. The resulting residue was re-dissolved in a solution consisting of (methanol: acetonitrile: water, 1:1:1) mixed with isopropanol (IPA) in a ratio of 1:1, followed by centrifugation at 4℃ and 20,000 rpm for an additional 5 min. Finally, 180 µL of the supernatant was taken for LC-MS analysis.

### LC-MS conditions

Chromatographic parameters include the use of an ACQUITY UPLC^®^ BEH C18 column (100 × 2.1 mm, 1.7 μm). The mobile phase consists of A: acetonitrile and water in a ratio of 6:4 (with 10 mM ammonium acetate and 0.1% formic acid) and B: isopropanol, acetonitrile, and water in a ratio of 90:10:0.1 (also with 10 mM ammonium acetate and 0.1% formic acid). The reference conditions for gradient elution in liquid chromatography are as follows: The flow rate is maintained constant at 600 µL/min throughout the entire elution process. Between 0 and 1.5 min, the composition of mobile phase A is set at 68%, while mobile phase B constitutes 32%. From 1.5 to 15.5 min, the percentage of mobile phase A decreases linearly from 68% to 15%, and correspondingly, the proportion of mobile phase B increases linearly from 32% to 85%. Between 15.5 and 15.6 min, the concentration of mobile phase A rapidly decreases to 3%, while that of mobile phase B increases to 97%. From 15.6 to 18 min, the composition remains stable at 3% for mobile phase A and 97% for mobile phase B. Between 18 and 18.1 min, the proportion of mobile phase A quickly returns to 68%, with mobile phase B decreasing to 32%. Finally, from 18.1 to 21 min, the composition of mobile phase A is maintained at 68%, and mobile phase B remains at 32%. The flow rate is set at 0.6 ml/min, with an injection volume of 10 µL, and the column temperature maintained at 45℃.

Mass spectrometry was conducted using a triple quadrupole tandem mass spectrometer (Triple Quad 7500 from Sciex Inc.) equipped with an electrospray ionization (ESI) source. In positive ion mode, the parameters were as follows: spray voltage at 5000 V, nebulizer pressure at 60 psi, auxiliary gas pressure at 70 psi, curtain gas pressure at 48 psi, collision cell pressure at 7 psi, and ion source temperature maintained at 450 °C. For negative ion mode, the settings included a spray voltage of -4500 V, nebulizer pressure of 55 psi, auxiliary gas pressure of 70 psi, curtain gas pressure of 48 psi, collision cell pressure of 7 psi, and an ion source temperature also set to 450 °C.

### Lipidomics data analysis

The original metabolic data were processed using the Analyst 1.7.1 software package to systematically collect and organize pertinent information, including chromatographic peak indices, sample names, peak intensity areas, retention times, and precise mass-to-charge ratios. Automated batch processing for tasks such as peak extraction, alignment, identification, and area integration was performed using MRMPROBS. Additionally, the raw metabolic data matrix was imported into the MetaboAnalyst 5.0 online tool (metaboanalyst.ca/faces/home.xhtml) for normalization, handling of missing values, and scaling to generate a processed metabolic data matrix that encompasses mass-to-charge ratios (m/z), retention times, and peak areas.

### Statistical analysis

This study employed SPSS 26.0 software for statistical analysis. Normally distributed continuous variables were expressed as mean ± standard deviation (Mean ± SD). Group comparisons were performed using independent-sample *t*-tests or Mann-Whitney *U* tests depending on whether the data met the assumption of normality. Categorical variables were presented as frequency and percentage (n, %), and group comparisons were conducted using the chi-square (*χ²*) test. For multivariate analysis, unsupervised principal component analysis (PCA) was initially applied to evaluate the overall sample clustering pattern, followed by supervised orthogonal partial least squares discriminant analysis (OPLS-DA) to identify metabolites that exhibited significant differences between groups. Core differential metabolites were selected based on the following criteria: variable importance in projection (VIP) score > 1 from the OPLS-DA model, adjusted *P*-value < 0.05 from univariate analysis, and |Log2FC| >1. Diagnostic performance was evaluated using ROC curve analysis. Structural identification of lipid molecules was carried out by referencing the Human Metabolome Database (HMDB). Pathway enrichment analysis was performed via the online platform MetaboAnalyst 5.0, using KEGG database annotations^21,22^, with an adjusted *P*-value < 0.05 (An FDR < 0.05 is the gold standard for controlling false positives in metabolomics and transcriptomics studies, effectively reducing the false positive bias caused by multiple statistical tests.) set as the threshold for statistically significant enrichment. A two-sided *P*-value < 0.05 was considered statistically significant.

## Results

### Differential analysis and functional enrichment analysis of IS and normal controls

**Fig. 2:**
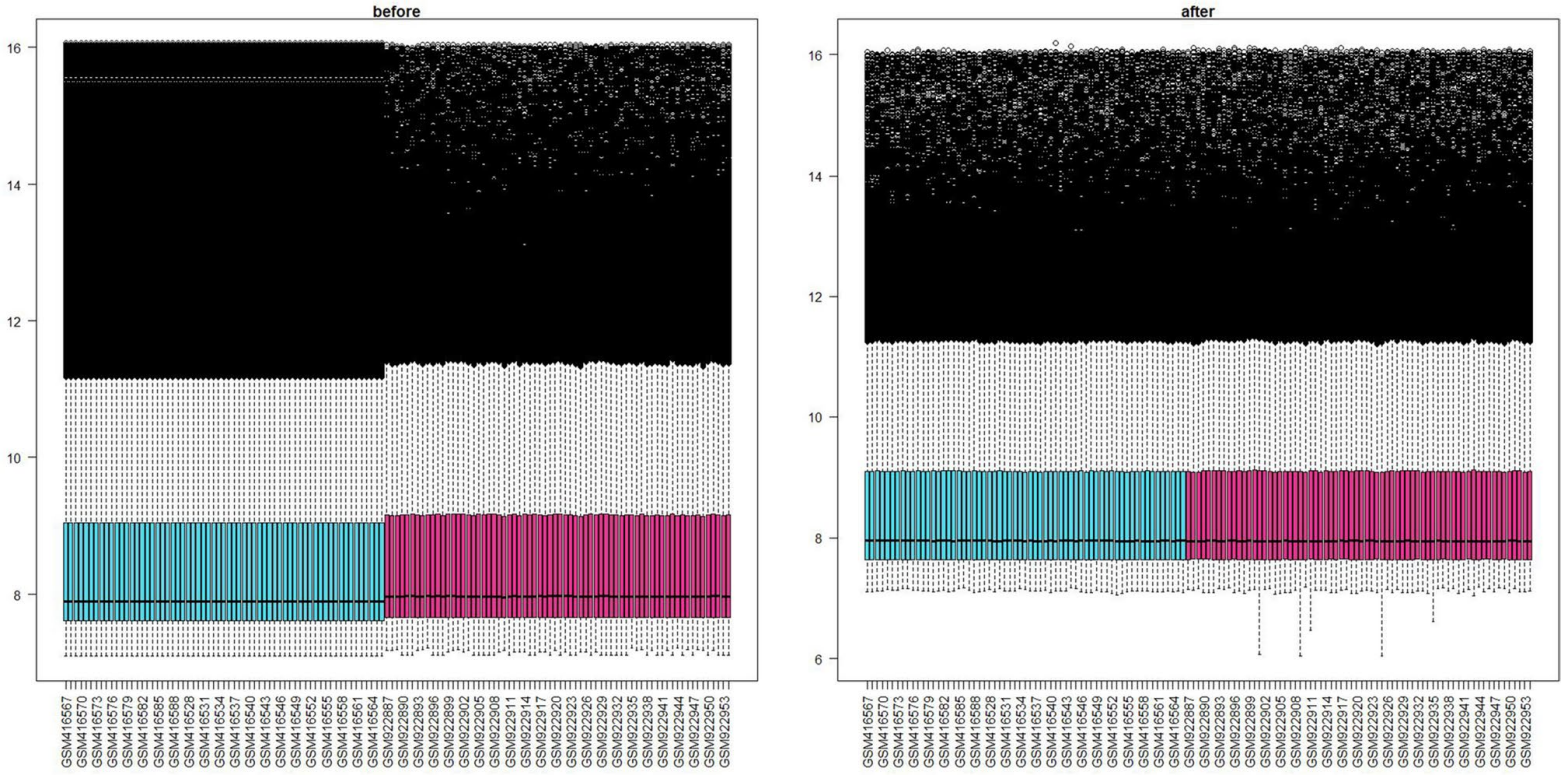
Comparison of two sets of data before and after de batching. The left panel illustrates the data distribution prior to correction, where distinct separation between the two datasets is evident, indicating a pronounced batch effect. The right panel displays the data distribution following correction, showing improved integration and substantial reduction of the batch effect, with samples clustering more consistently across groups.

In this study, peripheral blood gene expression data from IS patients and healthy controls were obtained from the GEO database (https://www.ncbi.nlm.nih.gov/geo/), and two datasets (GSE16561 and GSE37587) were selected based on predefined study criteria for subsequent analysis. Dataset GSE16561, generated using the GPL6883 platform, includes samples from 39 IS patients and 24 healthy controls^23^. Dataset GSE37587 contains gene expression profiles from 34 IS patients at both 24 h and 48 h post-onset; for this study, data collected at the 24-hour time point were used^24^. The R software was utilized to integrate two datasets derived from the same platform but distinct cohorts. To mitigate technical variability, batch effects were adjusted using the “SVA R package” ^14^. Subsequently, differential expression analysis was conducted using the “Limma R package” ^15^, with significance thresholds defined as *P* < 0.05 and |log_2_FC|>1 ^16^. A comparison of data distribution before and after batch correction is illustrated in Fig. 2, demonstrating a marked improvement in dataset consistency following adjustment.

### Results of gene enrichment analysis

**Fig. 3:**
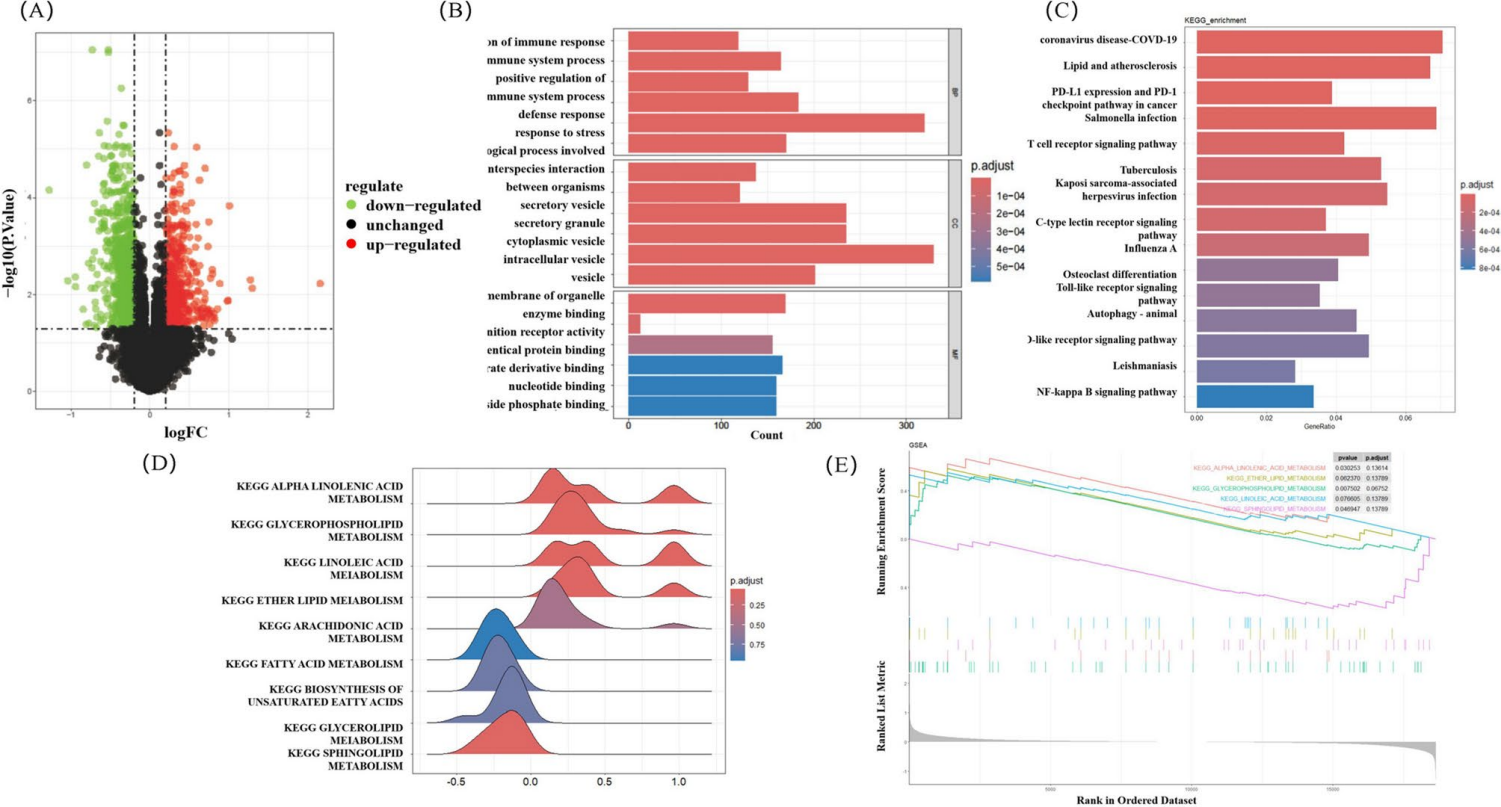
Bioinformatics analysis results. (**A**) Volcano plot of differentially expressed genes: The x-axis represents log₂ fold change (log_2_FC), and the y-axis represents -log₁₀-transformed *P*-values. In the IS group versus the HC group, red dots indicate up-regulated differentially expressed genes, blue dots indicate down-regulated differentially expressed genes, and gray dots represent non-significantly altered genes. The screening threshold was defined as log₂|FC| > 1 and *P* < 0.05, with the black dotted lines representing the corresponding cutoff values for log₂FC = ± 1 and *P* = 0.05, respectively. (**B**) GO enrichment analysis: The top 10 enriched terms in the BP, CC, and MF categories are displayed. The x-axis shows the number of enriched genes, and the y-axis lists the GO term names. Blue represents BP, red represents CC, and green represents MF; the color intensity reflects the level of significance (*P* value), with darker shades indicating smaller *P* values (*P* < 0.05). (**C**) KEGG pathway enrichment analysis: The x-axis represents the number of enriched genes, and the y-axis denotes the KEGG pathway names. Color intensity reflects the significance level, with darker shades corresponding to lower *P*-values. (**D**) Ridge plot of lipid metabolism pathways: This plot illustrates the distribution of gene set enrichment scores across lipid metabolism-related pathways. The color transitions gradually from blue, representing low values (i.e., low enrichment scores), to red, representing high values (i.e., high enrichment scores). A darker or more intense red hue indicates a higher standardized enrichment score for the corresponding pathway. (**E**) GSEA plot: Enrichment curves for five significantly down-regulated lipid metabolism pathways are shown. The x-axis represents the ranked list of genes based on expression changes, and the y-axis displays the running enrichment score. The distinct colored curves at the top represent different biological pathways; each vertical line indicates the position of the “target molecule” within the corresponding pathway’s molecular set in the ranked list, with the color of the line matching that of the respective top curve. The gray gradient area at the bottom reflects the direction of differential expression across the ranked molecules, where the intensity of the gradient corresponds to the magnitude of differential expression.

Differential expression analysis identified a total of 726 DEGs, comprising 485 up-regulated and 241 down-regulated genes. The volcano plot depicting these DEGs is presented in Fig. 3(A). The differentially expressed genes were analyzed for GO and KEGG pathway enrichment using the “clusterProfiler” R package^17^, with results presented in Fig. 3(B) and 3(C). GO enrichment encompassed three domains: biological process (BP), cellular component (CC), and molecular function (MF). In the BP category, differentially expressed genes were significantly enriched in processes including regulation of immune response, regulation of immune system process, defense response, and stress response, indicating a potential association between immune system dysregulation and the pathogenesis of IS. Regarding CC, the most highly enriched terms were secretory vesicles, secretory granules, and cytoplasmic vesicles, suggesting that these subcellular structures may play a critical role in IS-related transport mechanisms or signal transduction pathways. In the MF domain, the primary enriched functions included enzyme binding, pattern recognition receptor activity, and protein–protein interaction activity, implying significant alterations in molecular interactions during IS development. KEGG pathway analysis revealed the top five enriched pathways: Coronavirus Disease, Lipid and Atherosclerosis, PD-L1 Expression and PD-1 Checkpoint Pathway, Salmonella Infection, and T Cell Receptor Signaling Pathway. Notably, the enrichment of the Lipid and Atherosclerosis pathway provides a rationale for further investigation into the role of lipid metabolism in IS.

### GSEA analysis and hub genes related to lipid metabolism

Lipid metabolism-related KEGG gene sets, including those associated with sphingolipid metabolism, glycerophospholipid metabolism, glycerolipid metabolism, arachidonic acid metabolism, fatty acid metabolism, linoleic acid metabolism, and ether lipid metabolism, were retrieved from the MSigDB database (http://www.gsea-msigdb.org/gsea/msigdb). The enrichment levels of these gene sets within the differentially expressed genes of IS patients were assessed using GSEA^18^, with results presented in Fig. 3(D) and 3(E). Gene sets exhibiting |Normalized Enrichment Score (NES)|>1 (|NES|>1 indicates that the gene set shows a significant positive/negative enrichment trend.) and *P* < 0.05 were selected for further in-depth analysis. Five lipid metabolism-related gene sets—specifically, alpha-linolenic acid metabolism, linoleic acid metabolism, ether lipid metabolism, glycerophospholipid metabolism, and sphingolipid metabolism—were found to be significantly downregulated in the IS group. These findings suggest a potential suppression of lipid metabolic activity in IS patients, highlighting lipid metabolism dysregulation as a possible contributor to the pathogenesis of ischemic stroke and providing a foundation for future investigations.

Based on the bioinformatics analysis results from the GEO database, we have clearly identified that there are five core lipid metabolism pathways, including alpha-linolenic acid metabolism and glycerophospholipid metabolism, that are significantly downregulated in IS patients, providing preliminary molecular evidence for the pathological mechanism of IS-related lipid metabolism disorders. To further verify the expression characteristics of these pathways in clinical samples and screen lipid biomarkers with diagnostic value, this study adopts a case-control design. By recruiting IS patients and healthy controls and combining LC-MS lipidomics technology, we systematically detect the plasma lipid molecular profile, aiming to verify the association between lipid metabolism abnormalities and IS at the clinical level and simultaneously screen out a combination of core lipid markers with strong specificity and high diagnostic efficacy, providing clinically feasible detection targets for the early diagnosis of IS.

### Case-control analysis of traditional lipid factors

A total of 502 participants were enrolled in this study, comprising 251 individuals in the IS group with a mean age of 64.84 ± 12.16 years, and 251 healthy controls with a mean age of 62.86 ± 10.35 years. The two groups were well-matched with respect to age and gender distribution (*P* > 0.05). Detailed baseline characteristics are presented in Table 1. There were statistically significant differences in the distribution of type 2 diabetes, hypertension and obesity between the two groups (all *P* < 0.05), and there were also differences in medication use between the two groups (all *P* < 0.05). Additionally, the levels of TC, TG, and LDL-C were significantly elevated in the case group relative to the control group (all *P* < 0.05). In contrast, HDL-C levels were significantly lower in the case group than in the control group, and this difference was also statistically significant (*P* < 0.05).

**Table 1:** Basic characteristics of research subjects.

	Case groups (*n* = 251)	Control groups (*n* = 251)	χ^2^/Z value	*P* value
Gender, *n*(%)				
Male	153 (60.96)	98 (39.04)	0.135	0.713
Female	157 (62.55)	94 (37.45)		
Age(years)	64.53 ± 10.26	62.95 ± 8.79	1.854	0.064
Type 2 diabetes	70 (27.89)	18 (7.17)	37.259	< 0.001
Hypertension	140 (55.78)	38 (15.14)	90.561	< 0.001
Obesity	33 (13.15)	9 (3.59)	14.966	< 0.001
Medication status				
Antihypertensive drugs	89 (35.46)	22 (8.76)	51.922	< 0.001
Hypoglycemic drugs	46 (18.33)	11 (4.38)	24.244	< 0.001
TC(mmol/L)	4.82 ± 1.85	4.10 ± 0.81	5.668	< 0.001
TG(mmol/L)	1.84 ± 1.39	1.55 ± 0.94	2.094	0.037
LDL-C(mmol/L)	2.78 ± 0.92	2.38 ± 0.83	5.128	< 0.001
HDL-C(mmol/L)	1.05 ± 0.79	1.19 ± 0.73	2.074	0.039

### Lipidomics profiling results from the discovery cohort

**Fig. 4:**
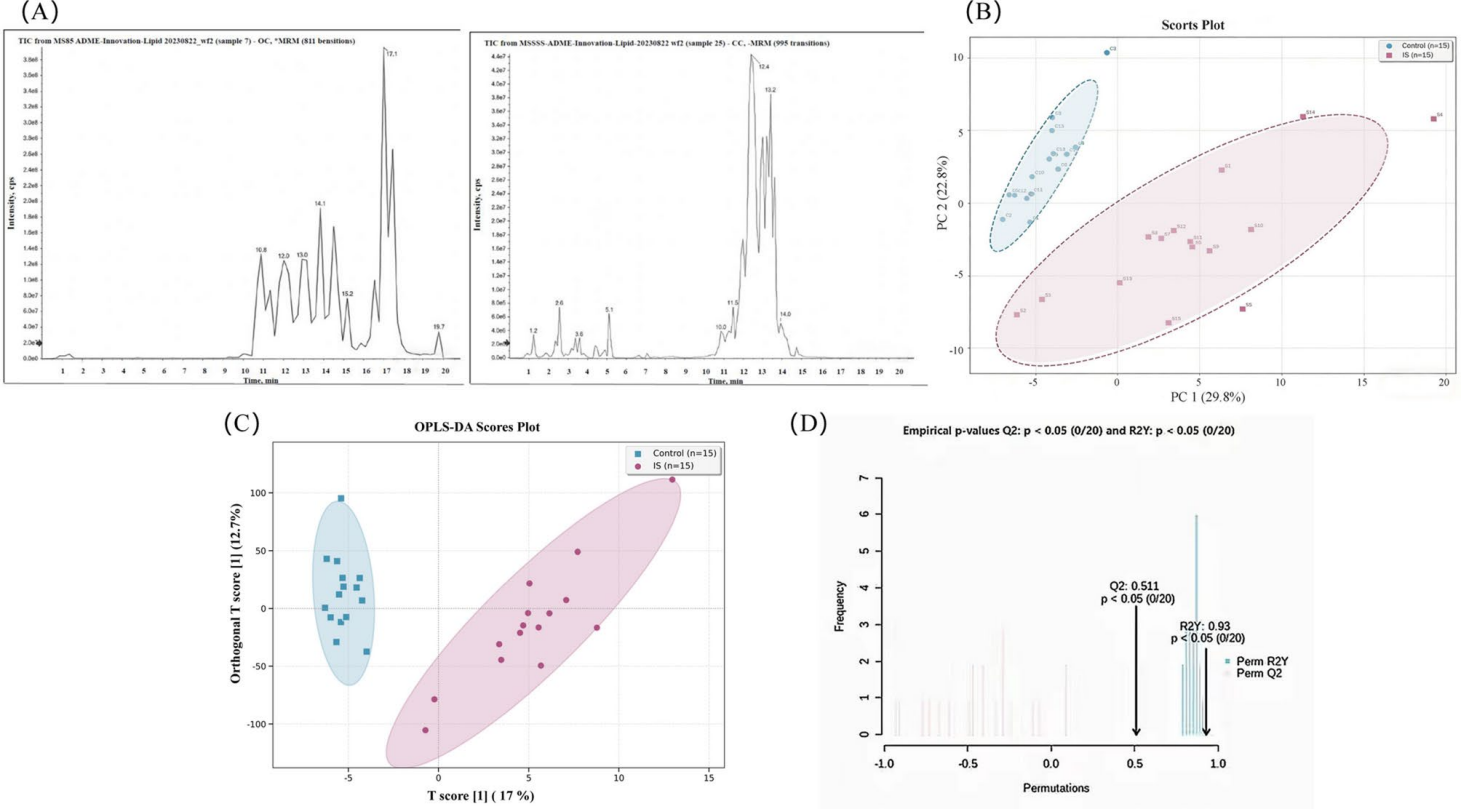
LC-MS detection and analysis results. (A) TIC chromatograms of QC samples in positive and negative ionization modes: the x-axis represents retention time (in minutes), and the y-axis represents ion intensity. The left panel illustrates the positive ion mode, while the right panel depicts the negative ion mode. The symmetric peak shapes and high degree of overlap indicate excellent analytical stability. (B) PCA score plot: The x-axis represents the first principal component (PC1, explaining 29.8% of the variance), and the y-axis represents the second principal component (PC2, explaining 22.8% of the variance). The red dots represent the IS group, and the blue dots represent the control group; the samples of the two groups show a partial separation trend in the PCA space, suggesting that there are inherent differences in the lipid metabolic profiles. (C) OPLS-DA score plot: The x-axis represents the predictive component t1 (explaining 17% of the variance), and the y-axis represents the orthogonal component t2 (explaining 12.7% of the variance). The red dots represent the IS group, and the blue dots represent the control group; the confidence ellipses are 95% confidence intervals (Hotelling T² ellipse). The two groups of samples are completely separated, indicating that the model can effectively distinguish between the IS. (D) Permutation test for model validation of OPLS-DA: The x-axis shows the number of permutations, and the y-axis shows R²Y (red) and Q² (blue). All Q² values of the permutations are lower than that of the original model, indicating that the model is robust and not overfitted.

Plasma samples from the discovery cohort were subjected to lipid separation and detection using the established lipid extraction protocol combined with LC-MS technology. The ion current chromatograms and mass spectrometry peak data of lipid molecules were obtained through LC-MS. The metabolomics data analysis software MRMPROBS developed by the Hiroshi Tsugawa team of the RIKEN Center for Sustainable Resource Science was used for automatic peak detection, peak alignment, peak identification and quantitative analysis. A total of 158 different lipid molecules were identified. The total ion chromatogram (TIC) of the quality control (QC) samples is shown in Fig. 4 (A). The chromatographic peaks of the QC samples have a high degree of overlap and a stable baseline, indicating that the detection system is stable and the data is reliable.

### Multivariate statistical analysis of lipidomics data

To evaluate global differences in lipid metabolic profiles between IS patients and healthy controls, this study employed unsupervised principal component analysis (PCA) followed by supervised orthogonal partial least squares discriminant analysis (OPLS-DA) for dimensionality reduction and group characterization. The Kaiser-Meyer-Olkin (KMO) measure was 0.827, indicating that the data were suitable for factor analysis. Bartlett’s test of sphericity yielded an approximate *χ²* value of 1081.816 (*P* < 0.001), indicating that the covariance matrix significantly deviated from the identity matrix and exhibited a significant correlation structure, thereby satisfying the prerequisites for PCA.

In the unsupervised PCA, the overall distribution of metabolic profiles revealed a natural clustering pattern between the two groups, as shown in Fig. 4(B). The first two principal components (PC1 and PC2) captured the major sources of variation in the lipidomics dataset. Samples from the healthy control group were predominantly located in the left half of the score plot, with a tightly clustered configuration, reflecting high internal consistency in their lipid metabolic profiles. In contrast, IS patient samples were primarily distributed in the right half, exhibiting greater dispersion. Despite this intra-group variability, the 95% confidence ellipses for the two groups showed only marginal overlap at the periphery, suggesting inherent differences in lipid metabolism between the groups. To enhance discrimination between groups, a supervised OPLS-DA model was applied. As illustrated in Fig. 4(C), the model achieved clear separation between IS patients and healthy controls. The first predictive component accounted for 17.0% of the variance, along which the two groups were completely separated, with non-overlapping 95% confidence ellipses, confirming the lipid metabolic profile’s capacity to effectively distinguish disease status.

Furthermore, the first orthogonal component in the OPLS-DA model primarily captures biological variation unrelated to group classification. Within this dimension, samples from the healthy control group exhibit a highly concentrated distribution, whereas those from the IS group display a considerably broader dispersion, indicating notable inter-individual heterogeneity in lipid metabolism features that are independent of inter-group discriminatory patterns. Subsequent Spearman rank correlation analysis revealed that the scores of this orthogonal component were significantly positively correlated with stroke severity, as measured by the National Institutes of Health Stroke Scale (*r*_*s*_=0.571, *P* = 0.026), and with TOAST clinical subtypes (*r*_*s*_=0.640, *P* = 0.010). In contrast, no statistically significant associations were observed with age (*r*_*s*_=0.336, *P* = 0.220) or gender (*r*_*s*_=0.217, *P* = 0.438), suggesting that these demographic factors contribute minimally to the observed metabolic heterogeneity within the IS group. Instead, the severity of ischemic injury and underlying etiological subtypes appear to be the primary clinical determinants driving such variation. Importantly, the intra-group variability captured by this orthogonal component is distinct from the core lipid metabolic differences between the IS and healthy control groups, and thus does not compromise the model’s ability to efficiently identify markers differentiating the two groups. Model validation results, presented in Fig. 4(D), confirmed robustness. The model exhibited strong explanatory power (R²Y = 0.93) and acceptable predictive ability (Q²=0.511). Permutation testing (*n* = 200) demonstrated that all permuted Q² values were lower than the original model’s Q², thereby ruling out overfitting and affirming the statistical validity and stability of the OPLS-DA model in discriminating between patient and control groups.

**Fig. 5:**
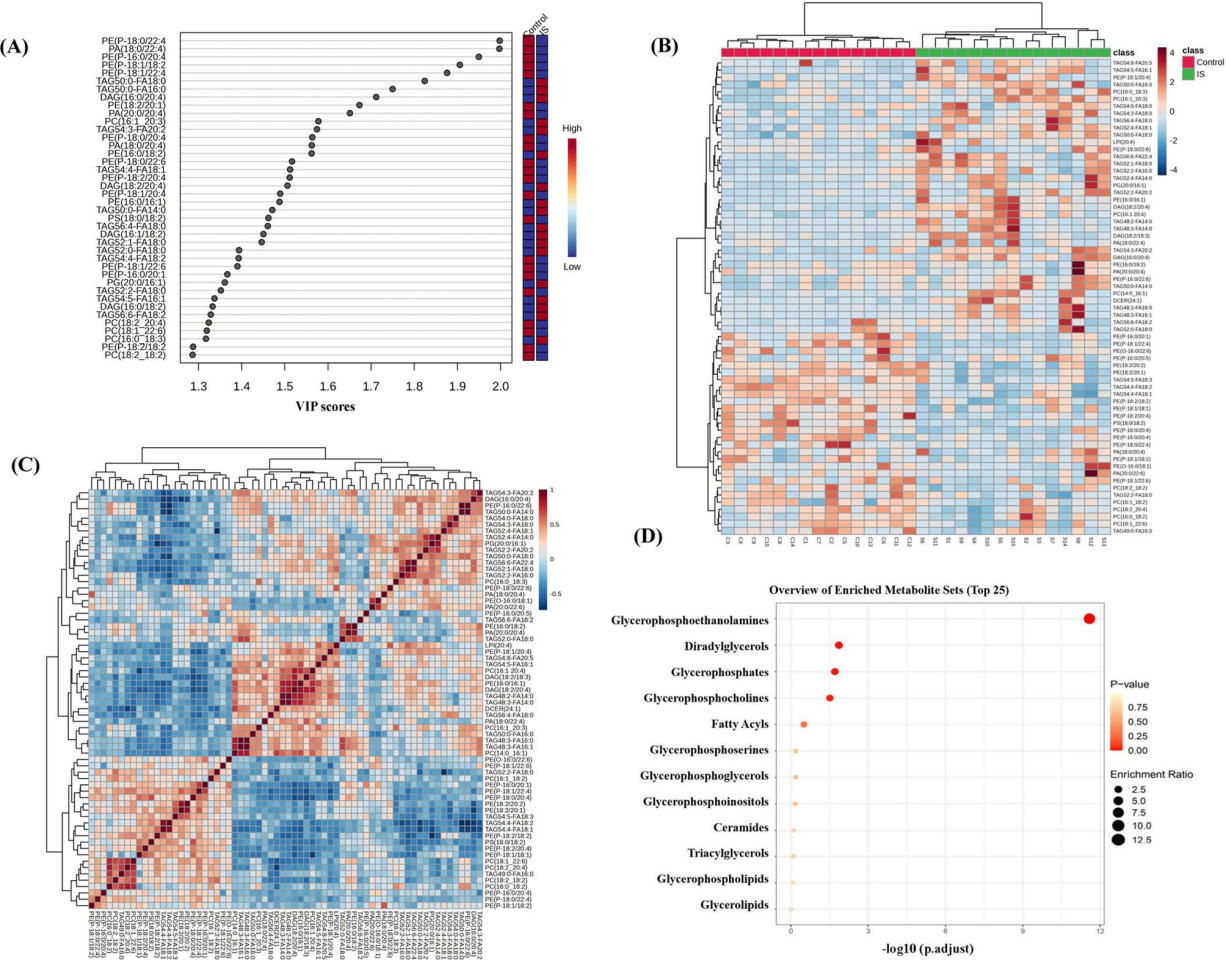
Analysis results of differentially expressed lipid molecules. (**A**) A horizontal bar chart of the top 40 VIP values of lipids: The y-axis represents lipid molecule identifiers, and the x-axis represents the VIP values of the OPLS-DA model (range: 1.300–1.998). Among them, 19 lipid VIPs were ≥ 1.5 (highly significant), and 21 lipid VIPs were 1.30 ≤ VIP < 1.5 (moderately significant). (**B**) Heatmap of differentially expressed lipid molecules: the x-axis represents sample identifiers (samples on the left correspond to the control group, and samples on the right correspond to the case group), the y-axis lists the names of the differentially expressed lipid molecules, and the color gradient reflects the expression levels, with red indicating higher expression and blue indicating lower expression. (**C**) Correlation coefficient matrix of 66 differentially expressed lipid molecules: both axes represent the names of lipid molecules, and the color intensity represents the correlation coefficient, where red denotes positive correlation and blue denotes negative correlation, with darker shades indicating stronger correlations. (**D**) KEGG pathway enrichment analysis of differentially expressed lipid molecules: the x-axis shows the enrichment ratio (number of enriched genes divided by the background gene count), the y-axis displays lipid metabolism pathways or categories, color intensity reflects the significance level (darker colors indicate smaller *P* values), and the size of each bubble corresponds to the number of enriched genes.

A total of 66 differentially expressed lipid molecules were identified based on the following criteria: VIP > 1 from the OPLS-DA model, univariate *t*-test significance (*P* < 0.05), and |Log₂FC|>1. The distribution of VIP values across lipid molecules is shown in Fig. 5(A), where higher VIP values indicate greater contribution to group classification. Heatmap analysis of these differential lipid molecules is displayed in Fig. 5(B). The color gradient visually represents relative expression levels—red denotes above-average expression, while blue indicates below-average expression. The heatmap reveals distinct expression patterns between the IS and control groups, further supporting significant alterations in lipid metabolism associated with IS.

Correlation analysis among the 66 differential lipid molecules was conducted, with results shown in Fig. 5(C). The sign and magnitude of the correlation coefficients reflect potential biological relationships: positive correlations may suggest synergistic interactions or shared regulatory pathways, whereas negative correlations could indicate competitive or inhibitory mechanisms. For instance, phosphatidylethanolamine (PE) species predominantly exhibit positive inter-molecular correlations, while diacylglycerol (DAG) species show negative correlations with certain PE molecules. This observed correlation structure provides valuable insights for constructing and interpreting the underlying lipid metabolic network. The 66 differential lipid molecules were annotated using the Human Metabolome Database (HMDB, https://hmdb.ca/), revealing that these compounds are primarily lipid-derived species, including PE, DAG and TAG, among others. KEGG pathway enrichment analysis was performed on the 66 differential lipid molecules, with results presented in Fig. 5(D). The most significantly enriched pathway was glycerophospholipid metabolism, followed by pathways associated with sphingolipid metabolism, fatty acid metabolism, and other related metabolic processes.

Further refinement based on |Log₂FC|>1 led to the identification of 19 key differentially expressed lipid molecules, detailed in Table 2. Among these, thirteen were downregulated, six were upregulated in the IS group. These molecules represent potential lipid biomarkers associated with IS pathophysiology.

**Table 2:** Significant differences in lipids between the IS and Control groups.

Lipids	m/z	RT (min)	log_2_(FC)	VIP	*P* value
PE(16:0/16:1)	688.49	12.09	-2.52	1.49	< 0.001
DAG(16:0/20:4)	634.50	13.37	-2.16	1.71	< 0.001
TAG48:3-FA16:0	810.53	16.64	-1.81	1.07	< 0.001
PE(P-18:0/22:4)	778.60	13.93	1.77	2.00	< 0.001
TAG48:3-FA14:0	810.53	16.54	-1.76	1.04	< 0.001
PA(18:0/22:4)	751.53	13.21	1.67	2.00	< 0.001
TAG48:3-FA16:1	812.53	17.10	-1.55	1.11	< 0.001
TAG48:2-FA14:0	810.53	16.99	-1.49	1.04	< 0.001
PG(20:0/16:1)	775.55	10.69	-1.29	1.36	< 0.001
DCER(24:1)	650.90	15.35	-1.26	1.23	< 0.001
TAG54:3-FA20:2	818.53	17.44	-1.17	1.57	< 0.001
PE(P-18:1/22:4)	776.60	13.11	1.15	1.88	< 0.001
DAG(18:2/20:4)	658.50	12.46	-1.10	1.51	< 0.001
DAG(16:1/18:2)	608.50	12.69	-1.09	1.45	< 0.001
PE(P-18:1/18:2)	724.50	12.70	1.08	1.91	< 0.001
PE(P-16:0/20:5)	720.50	11.78	1.08	1.13	< 0.001
PC(14:0/16:1)	704.55	10.65	-1.07	1.17	< 0.001
TAG56:4-FA18:0	820.53	17.68	-1.06	1.46	< 0.001
PE(P-18:0/22:6)	774.50	13.11	1.034	1.52	< 0.001

Note: The log₂FC was determined based on the ratio of lipid abundance in IS group relative to the healthy control group. A positive log₂FC value indicates upregulation of the lipid species in the IS group, whereas a negative value indicates downregulation.

### Results from the internal validation cohort excluding individuals with comorbidities

Following the exclusion of IS patients with T2D ( *n* = 70), hypertension (*n* = 140), or obesity (*n* = 33), a subgroup of 111 IS patients free of comorbidities was established. Similarly, in the control group, individuals with T2D (*n* = 18), hypertension (*n* = 38), or obesity (*n* = 9) were excluded, resulting in a comorbidity-free control cohort of 213 participants. Plasma lipid profiles were subsequently re-analyzed and compared between the two groups. Of the 19 lipids initially identified as differentially expressed, 15 remained statistically significant (VIP > 1, *P* < 0.05) in this comorbidity-free subset (see Table 3). Notably, the decreased levels of PE (P-18:0/22:4) and PE (P-18:0/22:6), along with the increased levels of DCER (24:1) and DAG (16:0/20:4), were consistently observed in both the overall cohort and the comorbidity-free subgroup.

**Table 3:** Significant differences in lipids between the IS (*n* = 111) and Control (*n* = 213) groups.

Lipids	m/z	RT (min)	log_2_(FC)	VIP	*P* value
PE(16:0/16:1)	688.49	12.09	-2.18	1.32	< 0.001
DAG(16:0/20:4)	634.50	13.37	-2.75	2.28	< 0.001
TAG48:3-FA16:0	810.53	16.64	-1.55	1.05	< 0.001
PE(P-18:0/22:4)	778.60	13.93	2.35	2.45	< 0.001
TAG48:3-FA14:0	810.53	16.54	-1.48	1.02	< 0.001
PA(18:0/22:4)	751.53	13.21	1.42	1.85	< 0.001
TAG48:3-FA16:1	812.53	17.10	-1.32	1.08	< 0.001
TAG48:2-FA14:0	810.53	16.99	-1.27	1.03	< 0.001
PG(20:0/16:1)	775.55	10.69	-1.16	1.21	< 0.001
DCER(24:1)	650.90	15.35	-1.82	1.67	< 0.001
TAG54:3-FA20:2	818.53	17.44	-1.05	1.42	< 0.001
PE(P-18:1/22:4)	776.60	13.11	1.02	1.78	< 0.001
DAG(18:2/20:4)	658.50	12.46	-0.98	1.35	< 0.001
DAG(16:1/18:2)	608.50	12.69	-0.92	1.28	< 0.001
PE(P-18:0/22:6)	774.50	13.11	1.68	2.08	< 0.001

Note: The log₂FC was determined based on the ratio of lipid abundance in IS group relative to the healthy control group. A positive log₂FC value indicates upregulation of the lipid species in the IS group, whereas a negative value indicates downregulation.

### Analysis of the diagnostic efficacy of differentially expressed lipid molecules for IS

Correlation among 15 significantly differentially expressed lipid molecules was evaluated to determine the suitability for principal component analysis. The KMO measure of sampling adequacy was 0.649, and Bartlett’s test of sphericity yielded a *χ²* value of 669.427 (*P* < 0.001), indicating a moderate correlation structure among variables and confirming that the dataset is appropriate for PCA-based dimensionality reduction. Principal components with eigenvalues greater than 1 were retained in the PCA. Factor loadings of each lipid molecule on the extracted components were computed, and principal component score coefficients were derived by dividing these loadings by the square root of the corresponding eigenvalue. Subsequently, normalized weighted averages of the score coefficients were calculated to determine the relative weights of the lipid molecules, with results presented in Table 4.

**Table 4:** Indicator Weights.

Differential lipid molecules	Comprehensive score model coefficient	Weight of index
PE(P-18:1/22:4)	0.231	0.177
PE(16:0/16:1)	0.220	0.168
TAG48:3-FA16:1	0.045	0.035
DAG(18:2/20:4)	0.032	0.025
PE(P-18:0/22:6)	0.012	0.009
PE(P-18:0/22:4)	0.010	0.008
DCER(24:1)	0.010	0.008
TAG48:3-FA16:0	0.010	0.008
DAG(16:0/20:4)	0.010	0.007
PA(18:0/22:4)	0.009	0.007
DAG(16:1/18:2)	0.008	0.006
TAG48:2-FA14:0	0.007	0.005
TAG48:3-FA14:0	0.007	0.005
TAG54:3-FA20:2	0.006	0.004
PG(20:0/16:1)	0.005	0.004

**Fig. 6:**
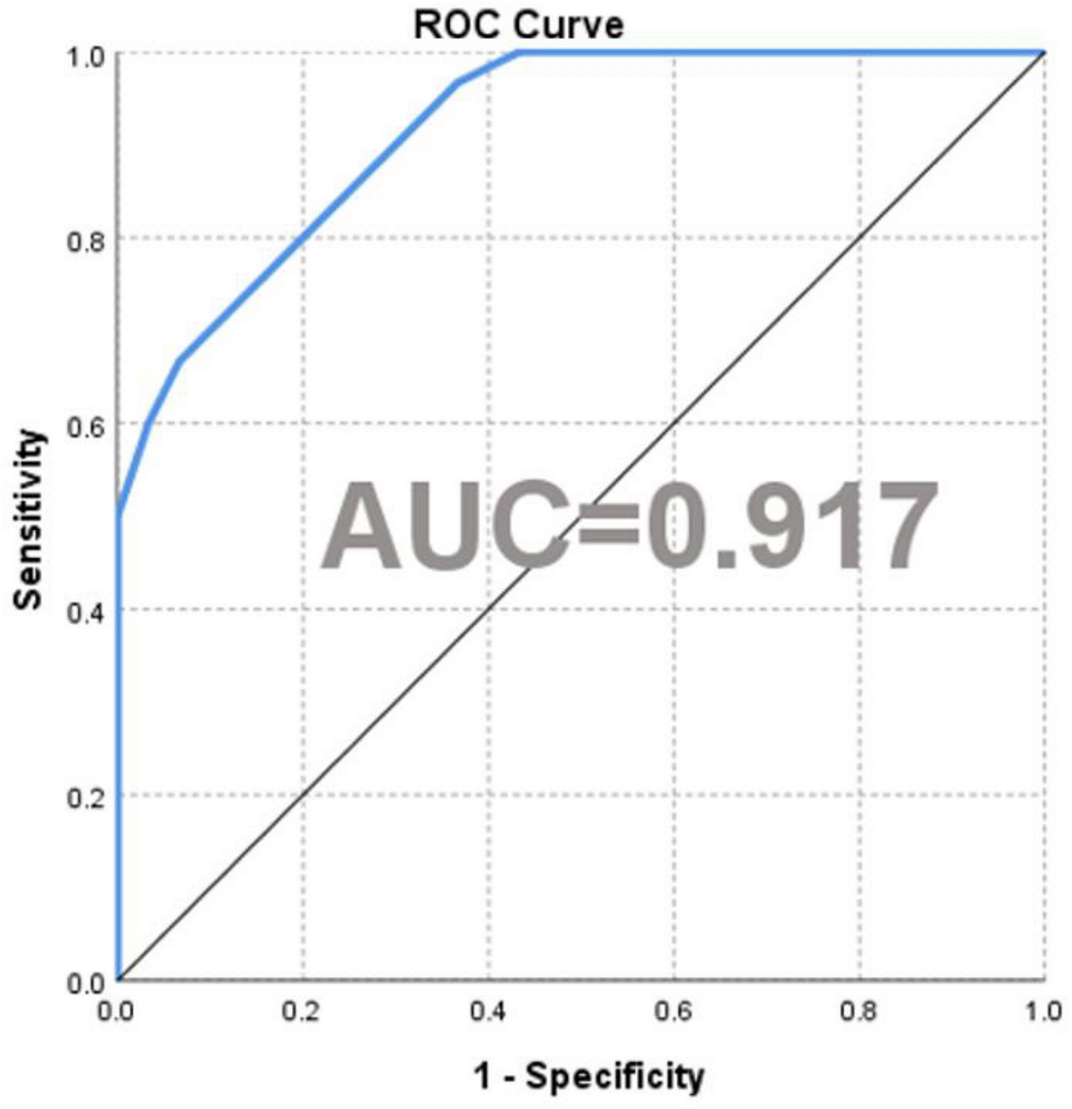
ROC Curve.

The five lipid molecules with the highest weights were PE (P-18:1/22:4), PE (P16:0/16:1), TAG48:3-FA16:1, DAG(18:2/20:4) and PE(P-18:0/22:6), collectively accounting for 84.654% of the total weight. This combination was identified as the core lipid biomarker panel for IS diagnosis. A diagnostic model was developed based on these five lipid molecules, and its performance was assessed using a receiver operating characteristic (ROC) curve. The AUC was 0.917 (95% confidence interval: 0.852–0.982), with a sensitivity of 60.0%, specificity of 96.7%, and a Youden index of 0.567, demonstrating strong diagnostic accuracy for IS, with results presented in Fig. 6.

## Discussion

With the continuous growth of the global aging population, the burden of stroke has become increasingly severe^25^. Compared to hemorrhagic stroke, IS is more prevalent^26^. The risk factors associated with the onset of ischemic stroke primarily consist of non-modifiable factors such as age and gender, as well as modifiable factors that can be influenced through lifestyle adjustments. The modifiable risk factors include hypertension, cardiovascular disease, dyslipidemia, diabetes, asymptomatic carotid artery stenosis, overweight and obesity, insufficient physical activity, unhealthy dietary habits and poor nutrition, smoking, and excessive alcohol consumption. Except for lifestyle-related behaviors, most of these risk factors are closely linked to metabolic dysregulation^27^. In recent years, numerous studies have investigated the association between IS and blood lipid profiles; however, the majority of these studies have primarily focused on conventional clinical lipid markers such as TG and TC^28^. Large-scale studies have consistently demonstrated that elevated levels of these lipid parameters are generally associated with adverse clinical outcomes^29^. Although a considerable number of domestic and international studies have explored the relationship between blood lipid parameters and stroke, the interpretation and analysis of their role in stroke pathogenesis remain insufficiently comprehensive.

The GSEA enrichment analysis of specific lipid metabolism pathway gene sets revealed that five lipid metabolism-related characteristic gene sets and signaling pathways were significantly downregulated in the IS group. Linolenic acid is an essential fatty acid for the human body. In nature, linolenic acid exists in two crystalline forms: α and γ. Among these, α-linolenic acid and γ-linolenic acid are the most common isomers^30^. Research has demonstrated that α-linolenic acid, as an omega-3 polyunsaturated fatty acid, exerts neuroprotective effects. Conversely, a severe deficiency in omega-3 fatty acid intake may increase brain vulnerability and serve as a significant risk factor for the onset and progression of neurological disorders^31,32^. Genes involved in linoleic acid metabolism and α-linolenic acid metabolism are downregulated in patients with IS, potentially contributing to an unfavorable prognosis.

Glycerophospholipids represent the most abundant class of membrane lipids, consisting of a glycerol backbone, two fatty acid chains, and a polar head group. In human cells, they primarily give rise to phospholipids such as phosphatidylserine (PS), phosphatidylcholine (PC), phosphatidylethanolamine (PE), phosphatidylglycerol (PG), and phosphatidylinositol (PI)^33^. The occurrence of ischemic stroke can lead to cerebral circulatory disturbances, which may disrupt phospholipid metabolism. Numerous studies on lipid metabolism in ischemic stroke have reported significant differences in glycerophospholipid metabolism between ischemic stroke patients and control groups^34–36^. Moreover, evidence suggests that the development of post-stroke depression is associated with reduced glycerophospholipid levels in the hippocampus^37^. A review on Alzheimer’s disease also highlighted the critical role of the glycerophospholipid metabolism pathway in maintaining neural function^38^. These findings indicate that the downregulation of glycerophospholipid metabolism-related genes in ischemic stroke patients is closely linked to disease onset and prognosis.

Ether lipids are essential constituents of cellular membranes and play a pivotal role in various biological processes, including cell signal transduction, apoptosis, and immune responses^39,40^. In recent years, accumulating evidence has demonstrated that dysregulation of ether lipid metabolism is closely associated with multiple metabolic disorders, such as diabetes, hyperlipidemia, and obesity^41,42^, all of which are well-established risk factors for IS. Sphingolipids, as fundamental structural components of eukaryotic cell membranes^43^, are particularly enriched in the central nervous system. Ceramide (Cer), serving as the central precursor for all complex sphingolipids—including sphingomyelin and glycosphingolipids—not only contributes to the structural integrity of the plasma membrane but also regulates intercellular interactions and recognition processes. Findings from numerous in vitro hypoxia models, animal studies, and clinical investigations consistently support a significant link between aberrant ceramide metabolism and the progression of cerebral ischemia^44^. Furthermore, elevated plasma ceramide levels have been reported in patients with large artery atherosclerotic stroke and cerebral small vessel disease^45^. Importantly, alterations in sphingolipid molecule levels are also correlated with stroke prognosis: reduced sphingolipid concentrations are frequently observed alongside unfavorable clinical outcomes^46,47^. In this study, GSEA analysis revealed that deoxyceramide (24:1), a distinct sphingolipid subtype, was significantly increased in the plasma of IS patients. This alteration was accompanied by downregulation of gene sets involved in both ether lipid and sphingolipid metabolic pathways. Collectively, these findings, along with evidence of functional pathway-level dysregulation, offer novel insights into the involvement of sphingolipid metabolism in the pathogenesis of IS.

Numerous studies have investigated the association between lipid levels and the onset of stroke. A majority of published literature indicates that abnormal lipid profiles constitute a risk factor for ischemic stroke^48–50^. However, our analysis of lipid parameters in this study revealed that elevated levels of TC, TG, and LDL-C may indeed increase the likelihood of disease occurrence, whereas HDL-C exhibits a protective effect. While dyslipidemia remains a significant risk factor for IS, not all elevated lipid parameters contribute to an increased risk.

The secondary pathophysiological responses elicited by cerebral ischemia involve a complex cascade, with neuronal depolarization serving as one of the pivotal initiating events. This process triggers the excessive release of excitatory amino acids, which subsequently activate their respective receptors and facilitate the opening of calcium ion (Ca^2+^) channels, leading to intracellular calcium overload. The elevated intracellular Ca^2+^ concentration activates multiple calcium-dependent enzyme systems, including phospholipase A_2_ (PLA_2_), phospholipase C (PLC), and phospholipase D (PLD)^51^. These enzymes significantly impact cell membrane integrity: PLA₂catalyzes the hydrolysis of membrane phospholipids—such as PC and PE—yielding DAG, PA, and arachidonic acid (ArAc). This reaction destabilizes the membrane structure and promotes the synthesis of inflammatory mediators, such as prostaglandins and leukotrienes, thereby exacerbating neural tissue injury. Activation of PLC initiates the phosphatidylinositol signaling pathway, generating DAG and inositol triphosphate, with DAG playing a central role in intracellular signal transduction. Similarly, PLD activation hydrolyzes phospholipids to produce DAG and PA, the latter of which can be further metabolized into ArAc—the precursor of prostaglandins^51^.

Research suggests that the severity of ischemic brain injury may precipitate secondary dysregulation of lipid metabolism. Disruption of the blood-brain barrier following stroke can initiate oxidative stress-mediated lipid peroxidation reactions^52^. Increased levels of oxidative stress biomarkers, including malondialdehyde (MDA) and 4-hydroxynonenal (4-HNE), are associated with direct neuronal cytotoxicity and correlate with higher National Institutes of Health Stroke Scale (NIHSS) scores^53^. These metabolic disturbances are secondary manifestations that reflect the extent of cerebral damage rather than intrinsic biological differences between IS patients and healthy individuals. Furthermore, the heterogeneity of ischemic stroke etiologies is captured by the TOAST classification system^54^. Distinct lipid metabolic profiles are observed across TOAST subtypes: large artery atherosclerosis (LAA) is commonly linked to pronounced abnormalities in cholesterol metabolism and intimal lipid accumulation^55^, whereas cardioembolic stroke (CE) is characterized by elevated triglyceride levels and reduced lysophosphatidylcholine concentrations^56^. Such subtype-specific metabolic alterations contribute to increased metabolic variability within the IS population.

The findings of this study provide direct evidence supporting these lipid-mediated molecular cascade reactions. First, a significant reduction in plasma levels of PE—a primary substrate for PLA_2_—was observed in patients with IS, including PE (P-18:0/22:4) (Log_2_(FC)=-2.35) and PE(P-18:0/22:6) (Log_2_(FC)=-1.68), consistent with enhanced PLA_2_ activity and increased PE hydrolysis during cerebral ischemia. Second, levels of DAG—a common product of PLA_2_, PLC, and PLD—were elevated (e.g., DAG(16:0/20:4), Log_2_(FC) = 2.75; DAG(18:2/20:4), Log_2_(FC) = 0.98), indicating overall upregulation of phospholipase activity. Third, PA levels exhibited an upward trend (PA(18:0/22:4), Log_2_(FC) = 1.42), further corroborating the activation of this signaling pathway. Additionally, prior studies have demonstrated that PE holds considerable potential for use in the diagnosis and prognosis assessment of IS^52,53^.

This study highlights the association between lipid metabolism disorders and the occurrence of IS; however, it remains unclear whether the former serves as a causal factor or represents a secondary consequence of the latter. IS may, through mechanisms such as inducing inflammatory responses, oxidative stress, and cellular damage, subsequently influence lipid metabolism^54–56^. For example, neuroinflammation and oxidative stress are rapidly activated following IS and are closely linked to dynamic alterations in lipid metabolites^57,58^. Moreover, ischemia-reperfusion injury can trigger cell death, leading to the release of intracellular components and modifying both local and systemic lipid microenvironments^59,60^. Conversely, dysregulated lipid metabolism may preexist prior to IS onset, potentially acting as a risk factor in disease initiation and progression^61,62^. Notably, PLA2 is recognized as an independent risk marker in the inflammatory pathway of atherosclerosis, with substantial evidence supporting its role in coronary heart disease; however, its specific involvement in cerebrovascular diseases requires further investigation^61^. Therefore, establishing a causal relationship between lipid metabolism disturbances and IS necessitates more comprehensive longitudinal analyses and interventional studies.

Elevated levels of TG, TC, and LDL-C are frequently associated with comorbid conditions such as T2D, hypertension, and obesity—all of which are known to significantly influence lipid metabolism^49,50^. To mitigate the confounding effects of these variables, a sensitivity analysis was conducted, excluding patients with T2D, hypertension, or obesity, resulting in the formation of a “no comorbidity” subgroup comprising IS patients and healthy controls. Re-comparison of plasma lipid profiles between these groups revealed that 15 lipid species remained statistically significant. Notably, the decreased levels of PE(P-18:0/22:4) and PE(P-18:0/22:6), along with increased levels of DCER(24:1) and DAG(16:0/20:4), were consistently observed in both the full cohort and the no-comorbidity subset. The consistency of these expression trends across subgroups suggests that the differential regulation of PE(P-18:0/22:4), PE(P-18:0/22:6), DAG(16:0/20:4), and DCER(24:1) is independent of conventional metabolic comorbidities such as T2D, hypertension, and obesity. This strengthens the validity of these lipids as potential biomarkers for IS that are not confounded by traditional risk factors. Nevertheless, it should be acknowledged that residual confounding—such as undiagnosed metabolic disturbances—cannot be entirely ruled out. Future research employing larger sample sizes is warranted to further validate these findings. Investigating the utility of lipid metabolites as biomarkers holds substantial promise for improving early detection, guiding intervention strategies, and enhancing prognostic outcomes in ischemic stroke.

This study, through systematic lipidomics analysis, successfully identified and validated a five-lipid biomarker panel with high diagnostic accuracy for IS. IS constitutes the majority of all stroke cases and ranks as the second leading cause of global mortality. Given its rapid progression, early and accurate diagnosis is critical^62^. However, conventional lipidomics workflows based on LC-MS platforms and standard analytical protocols are time-intensive, posing a significant technical barrier to the real-time application of this biomarker panel in hyperacute clinical decision-making, such as intravenous rt-PA thrombolysis. This limitation in analytical turnaround time represents both a key constraint of the current study and a major challenge for clinical translation^63^. Nevertheless, the primary contribution of this work lies in establishing well-defined molecular targets and a foundational framework for addressing this challenge.

To enable the rapid deployment of this biomarker panel in emergency settings, future translational efforts should focus on two strategic directions:

First, methodological optimization for accelerated analysis is essential. This includes developing rapid extraction protocols tailored to the five target lipids and implementing targeted mass spectrometry approaches, such as multiple reaction monitoring (MRM) using triple quadrupole mass spectrometry (TQ-MS)^64,65^. MRM enables selective detection of specific precursor-product ion transitions, allowing highly sensitive and specific quantification of target analytes in complex biological matrices^66^. These advancements are expected to reduce assay time from several hours to within minutes^66^, thereby aligning with the temporal demands of acute stroke diagnosis.

Second, iterative integration and development of advanced technical platforms are required. Specifically, coupling this streamlined detection approach with point-of-care testing (POCT) technologies^67^—such as miniaturized mass spectrometers or aptamer-based microfluidic devices^67^—could facilitate “sample-in, result-out” diagnostics at the bedside or in emergency departments^68^. Such integration would empower clinicians to make timely, individualized treatment decisions within the narrow therapeutic window following stroke onset. Moreover, it holds transformative potential by shifting the paradigm of ischemic stroke diagnosis from centralized laboratory testing to immediate, decentralized bedside assessment, thereby improving both the speed and precision of hyperacute stroke management.

In summary, dysregulation of lipid metabolism is closely implicated in ischemic stroke. The present study indicates that perturbations in five key lipid metabolic pathways—namely linolenic acid metabolism, glycerophospholipid metabolism, ether lipid metabolism, sphingolipid metabolism, and phospholipase-mediated lipid hydrolysis—may play critical roles in the onset and progression of IS. While elevated TC, TG, LDL-C, and reduced HDL-C are well-established independent risk factors for IS, this study identifies 15 novel differentially expressed lipid species—including specific PE variants and DCER(24:1)—that may serve as promising candidates for further investigation as disease-specific biomarkers.

Limitations.

This study systematically analyzed the association between IS and lipid metabolism, providing valuable insights into the pathogenesis and clinical diagnosis of IS. However, certain limitations remain, which need to be addressed in future research. First, the inference of causal relationships is limited. As a case-control study, this research primarily revealed the correlation between lipid metabolism dysfunction and the occurrence of IS, but it cannot determine whether lipid metabolic disturbances are the cause of IS or secondary changes during the disease process. The stress response and tissue damage following IS may also exert reverse effects on lipid metabolism. Therefore, prospective cohort studies or interventional experiments are needed to further validate the causal relationships. Second, the study sample was derived from a single center, which may limit the generalizability of the findings. Future research should aim to expand the sample size, with a focus on including patients with IS at different time windows such as the ultra-early stage, acute stage, and recovery stage, to cover a more comprehensive clinical heterogeneity and optimize the model’s adaptability to patients at different stages. At the same time, a combined diagnostic model of “lipid markers + clinical risk factors” will be constructed by integrating clinical risk factors such as hypertension and dyslipidemia, effectively enhancing the overall sensitivity of the model through multi-dimensional feature fusion. Additionally, multi-center, large-sample external independent validation studies will be conducted to further calibrate the model threshold and improve its generalization ability and stability in real clinical scenarios. Moreover, functional experiments will be combined to deeply explore the potential mechanism of core lipid molecules in the pathogenesis of IS, and a comprehensive assessment system for the clinical applicability of biomarkers will be improved, comprehensively promoting the process of transforming this lipid diagnostic model from basic research to clinical application.

## Data Availability

The data used in this study are freely available on the database of GEO (https://identifiers.org/geo: GSE16561, https://identifiers.org/geo: GSE37587). Our analyses, protocols, and raw figures or other information related to this study could be requested from the corresponding author upon reasonable request.
